# Location and expression of Juno in mice oocytes during maturation

**DOI:** 10.5935/1518-0557.20170065

**Published:** 2017

**Authors:** Banri Suzuki, Yukou Sugano, Jun Ito, Haruka Saito, Sueo Niimura, Hideaki Yamashiro

**Affiliations:** 1 Laboratory of Animal Reproduction, Graduate School of Science and Technology, Niigata University, Japan

**Keywords:** Juno, knock down, oocyte-maturation

## Abstract

**Objective:**

Oocyte-sperm interaction is the essential step in fertilization. Juno, which has been known as Folate receptor 4, is the Izumo1 receptor expressed on the oocyte membrane. This study aims to investigate the location and expression of Juno in mice oocytes during maturation.

**Methods:**

To confirm the stage at which Juno expression begins in the mice oocytes and its location pattern, we performed immunostaining methods. Next, we evaluated Juno mRNA expression by a half quantitative RT-PCR. Juno knockdown oocytes were generated by microinjecting siRNA into the germinal vesicle (GV) stage oocytes, and analyzed the maturation rate.

**Results:**

Our results showed that Juno was expressed on the surface of the oocyte cytoplasmic membrane at the GV stage and it continues to be expressed at similar levels in the metaphase II (MII) stages of oocytes maturation. Interestingly, Juno is also expressed on the first polar body membrane at the MII stage. Fluorescence showing Juno expression was decreased in the oolemma of siRNA injected oocytes, but it was not completely disappearing in knock down oocytes. MII stage-rates of siRNA injected oocytes were not significantly different from sham controls.

**Conclusion:**

Juno was expressed in oocytes at the GV stage and it continues to be expressed at similar levels in later stages of oocytes maturation. Juno accumulation in oolemma during oocyte maturation is essential for fertilization, such as membrane recognition of both gametes.

## INTRODUCTION

To generate a normal diploid embryo, a sperm must penetrate the oocyte cytoplasm. Oocyte-sperm recognition and membrane fusion are crucial during mammalian fertilization (Okabe, 2013). Oocyte starts its maturation with germinal vesicle breakdown (GVBD), and nuclear maturation progresses to the stages of telophase I (TI), anaphase I (AI); and complete metaphase II (MII) oocytes can accept sperm in the cytoplasm (Fabritius *et al*., 2011).

Cluster of differentiation 9 (CD9), a molecule expressed on the oocyte surface, is a well-known factor required for membrane fusion between sperm and oolemma (Kaji *et al*., 2000; Miyado *et al*., 2000). CD9 defective female mice are infertile, as in the absence of CD9. In those mice, oocytes fail to fuse with sperm membrane, even though sperm bind the cytoplasmic membrane of oocytes (Kaji *et al*., 2000; Miyado *et al*., 2000). CD9 is expressed at the early stage of the oocyte growth, when the oocyte diameter is around 13-22 µm (Komorowski *et al*., 2006; Zyłkiewicz *et al*., 2010). Oocyte CD9 is enriched on the microvillar membrane, which is required for normal microvillar shape and distribution (Runge *et al.*, 2007). Therefore, CD9 knockout oocytes display functionally altered microvilli that are uniformly short (Runge *et al*., 2007). Since the sperm binds to the microvilli rich region of the oocyte, CD9 provides a platform for fusion between the sperm and the oocyte membranes (Runge *et al*., 2007).

In sperm, the counterpart of CD9 required for oocyte-sperm fusion is Izumo1 (Inoue *et al.*, 2005). In mice, Izumo1 first localizes to the acrosomal membrane of the ejaculated sperm and after the acrosome reaction it builds up at the equatorial segment of the sperm, pointing towards the possibility that the oocyte-sperm fusion begins in this region (Satouh *et al*., 2012). During the sperm-oocyte adhesion, Izumo1 interacts with the oocyte receptor, Juno (Bianchi *et al*., 2014). After binding of sperm protein Izumo1 and its egg receptor, Juno drives CD9 build up in the intercellular contact area prior to fusion during mammalian fertilization (Chalbi *et al*., 2014). Izumo1 carries a β-hairpin region that anchors two folded α-helix domains. Izumo1 is structurally stabilized after binding to Juno, as it brings about conformational change in the flexible Izumo1 β-hairpin region, which becomes elongated (Aydin *et al*., 2016; Melcher, 2016; Ohto *et al*., 2016). It has been suggested that binding to Juno promotes Izumo1 dimerization, subsequently preparing for oocyte-sperm membrane fusion (Inoue *et al*., 2015).

Juno is known as Folate receptor 4, which is expressed on the membrane of oocytes, via a C-terminal glycophosphatidylinositiol-anchor site (Jia *et al*., 2009; Bianchi *et al*., 2014). Female mice lacking Juno are infertile because sperm attachment to oocytes is prevented, although the oocytes appear morphologically normal (Bianchi *et al*., 2014). Juno is finally released into the perivitelline space immediately after the sperm binding, so that this receptor only contributes to oocyte-sperm recognition but not fusion (Bianchi *et al*., 2014). Unlike CD9, the expression pattern of Juno during oocyte maturation stages remains elusive.

This study aims to investigate the location and expression of Juno in mice oocytes during maturation.

## MATERIALS AND METHODS

### Animals and oocyte collection

Our study protocols followed the laboratory animal care guidelines, and all the procedures were conducted in accordance with the guidelines of the Ethics Committee for the Care and Use of Laboratory Animals for Research of Niigata University, Japan.

B6D2F1 female mice over 8-weeks old were used in this study. To collect GV stage oocytes, 5IU of pregnant mare serum gonadotropin (PMSG) (Calbiochem, La Jolla, CA, USA) were administered into the abdominal cavity of the mice. These mice were euthanized after 48h and the ovaries were excised. GV stage oocytes were collected from the antral follicles using a syringe, and then the oocytes were washed in 0.1% PVA-Leibovitz's L-15 medium (Invitrogen, Carlsbad, CA, USA). Next, to collect GVBD-TI, and MII stages oocytes, 5IU of PMSG and 5IU of human chorionic gonadotropin (hCG) (Calbiochem) were administered into the mice at 48h intervals. After 14-16 h, cumulus oocyte complex (COC) in the fallopian tube was flushed out using CZB medium containing 0.1% hyaluronidase (Sigma, St. Louis, MO, USA). The oocytes without cumulus cells were collected after washing with CZB medium.

### Oocytes fixation and staining

The oocytes were fixed in 10% buffer formalin solution for 30 min before overnight incubation with 500×LEAF Purified anti-mice FR4 (BioLegend, San Diego, CA, USA) primary antibody in a multidish at 4°C. The oocytes were then incubated with Alexa Flour 488 Goat anti-Rat IgG H&L (Abcam, Cambridge, UK) secondary antibody (500×) in the dark for 1h under constant rotation. Juno-stained oocytes were mounted on glass slides with 0.5-1.0 µl of ProLong Gold Antifade Reagent with DAPI (Invitrogen) - that stains the nucleus, and the stained oocytes were studied under confocal microscope (TCS SP8, Leica, Wetzlar, Germany). We categorized the fluorescence emanating such as strong or weak from Juno, as compared with controls in the same microscope settings, and calculated the percentage of oocytes in each category.

### RNA extraction and reverse transcription reaction

RNA was extracted from the oocytes using the Cells-to-cDNATM II Kit (Life Technologies, Carlsbad, CA, USA) as per the manufacturer's instructions. Thirty oocytes were transferred onto PCR tube and treated with 10 µl of Cell Lysis II Buffer followed by ultrasonication in ice. The lysed oocytes in the tube were incubated in water bath at 75°C for 10 min and then transferred to ice followed by incubation with 1 µl DNase at 37°C for 15 min, and then at 75°C for 5 min in a thermal cycler (BioRad, Hercules, CA, USA). RNA was quantitated by Nano-Drop (Thermo Fisher Scientific, Waltham, MA, USA) before synthesizing cDNA by 2.5 µl of RNA, 2 µl of dNTP Mix, 1 µl of Random Decamers, 2.5 µl of nuclease-free water (Takara, Siga, Japan) and incubating them first at 42°C for 30 min, followed by incubation at 92°C for 10 min.

### PCR reaction and Juno mRNA expression analysis

To measure the amount of Juno mRNA expression, we conducted a half quantitative RT-PCR using GAPDH as a positive control. The forward and reverse primers used for GAPDH detection were: 5′'-ACCACAGTCCATGCCATCAC-3′' and 5′'-TCCACCACCCTGTTGCTGTA-3′', respectively (Bonaconsa *et al.*, 2014). The primers for Juno mRNA were detection designed using NCBI (http://www.ncbi.nlm.nih.gov/) and Primer3Plus (http://frodo.wi.mit.edu/primer3/) sites, which were 5′'- CAACACATTCAAGGCCAGTC-3′' and 5′'-AGGAAATGTGGGTTGGAGAG-3′', respectively. Each PCR reaction solution was composed of 18.25 µl of RNase free water, 2.5 µl of 10xRT Buffer II, 1 µl of dNTP mixture, 0.25 µl of forward primer, 0.25 µl of reverse primer, 0.25 µl of Ex Taq (5IU/µl, Takara) and 2.5 µl of reverse transcriptase solution (total reaction volume was 25µl). The PCR was carried out in a thermal cycler (BioRad) for 40 cycles; each cycle comprised of a thermal denaturation at 95°C for 10 min, followed by an annealing reaction at 62°C for 2 min and finally an extension reaction at 72°C for 1 min. The amplified cRNA was resolved on agarose gel and the mRNA band was confirmed by LAS3000IR (FUJIFILM, Tokyo, Japan). The density of each mRNA band was measured by image analysis software, Image J, and the level of Juno mRNA expression in each oocyte maturation stage was calculated after equalization with the corresponding level of GAPDH mRNA.

### siRNA microinjection into GV stage oocyte

To generate Juno knockdown oocytes, we utilized the siRNA (Sigma) sequences targeting Juno mRNA, 5"-rCrCrCUUrGrCUrCUUUrArArCUUrCrATT-3" and 5"-UrGrArArGUUrArArArGrArGrCrArArGrGrGTT-3". The micromanipulator (DMIRB, Leica) equipped with the piezo (Prime Tech, Ibaraki, Japan) was used to inject siRNA into the GV stage oocytes. The siRNA at the concentrations of 10nM, 30nM and 50nM were prepared in Opti-MEM (Invitrogen). As a Sham control, Opti-MEM was injected into GV stage oocytes. Non-injected oocytes were used as control. After injection of several siRNAs, the GV stage oocytes were incubated in 5% FCS-Waymouth's MB752/1 medium (Invitrogen) for a day to allow its maturation into MII stage.

### Statistical analysis

The data was analyzed using variance analysis (ANOVA), followed by Tukey-Kramer tests. For all data, *p*<0.05 was considered significant. All analyses were conducted using StatView (Abacus Concepts Inc., Berkeley, CA, USA).

## RESULTS

### Juno expression pattern at different stages of oocyte maturation

**Figure 1:**
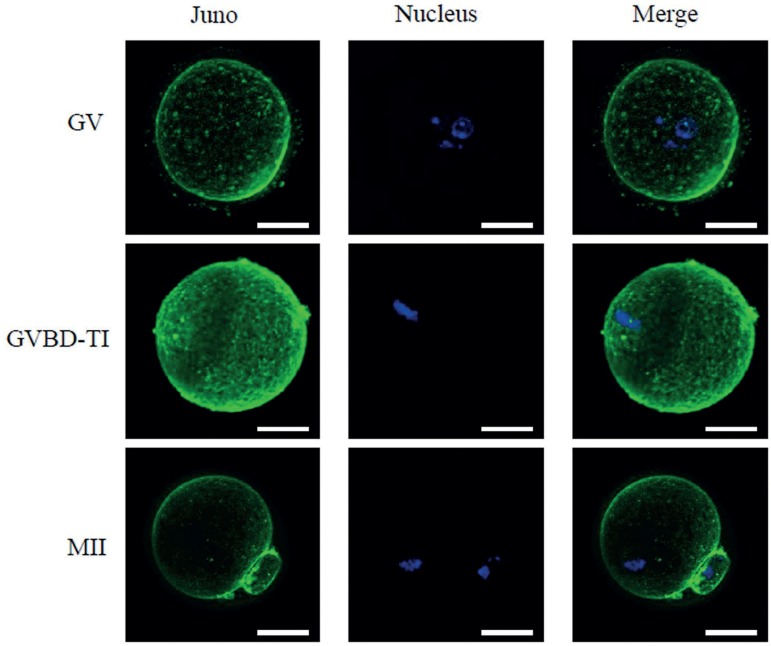
Immunofluorescence staining of immature oocytes in GV, GVBD-TI, and MII stages. Juno was stained in green and nucleus in blue. Scale bar=25 µm

**Figure 2:**
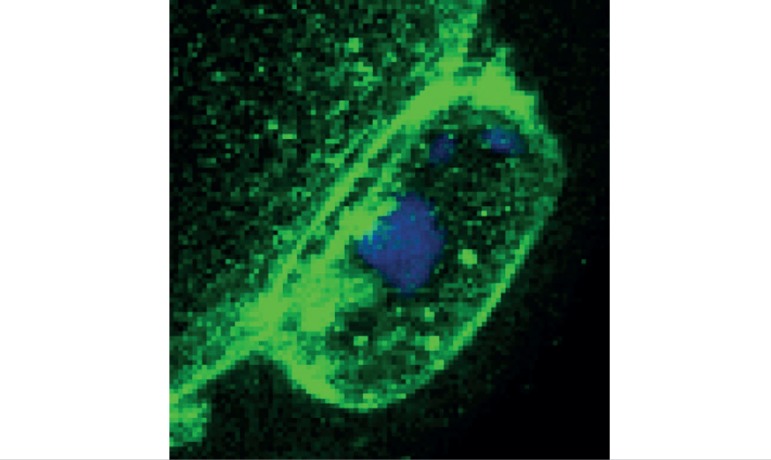
Immunofluorescence staining of the first polar body. Juno was stained in green and nucleus was in blue. Scale bar=5 µm

**Figure 3:**
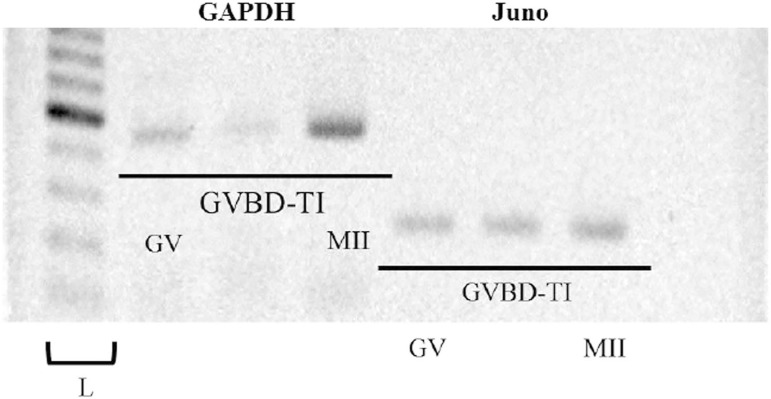
Electrophoretogram of Juno mRNA in GV, GVBD-TI, and MII stage oocytes. GAPDH serves as a control.

**Figure 4:**
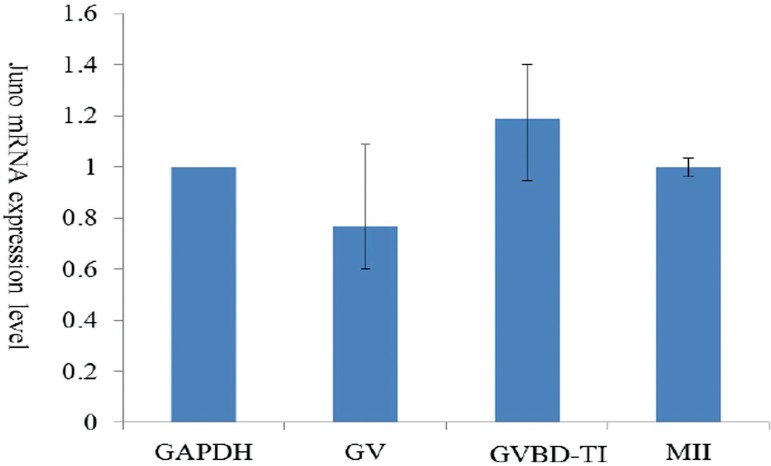
Juno mRNA expression in GV, GVBD-TI, and MII stage oocytes. Values represented as mean±SD of three replicate experiments.

To confirm the stage at which Juno expression begins in the oocytes and its location pattern, we performed immunostaining methods. As results, we observed that Juno is already expressed on oolemma at GV stage oocytes, and this expression pattern is consistent till the MII stage (Figure 1). Interestingly, Juno is also expressed on the first polar body membrane (Figure 2). Next, we evaluated the Juno mRNA expression by a half quantitative RT-PCR. The levels of Juno mRNA in GV, GVBD-TI, and MII stages after equalizing with GAPDH mRNA were 0.77, 1.19, and 1.00, respectively. Juno is expressed at similar levels throughout the different stages of oocyte maturation (Figures 3 and 4).

### Influence of Juno knockdown on maturation of oocytes

**Figure 5:**
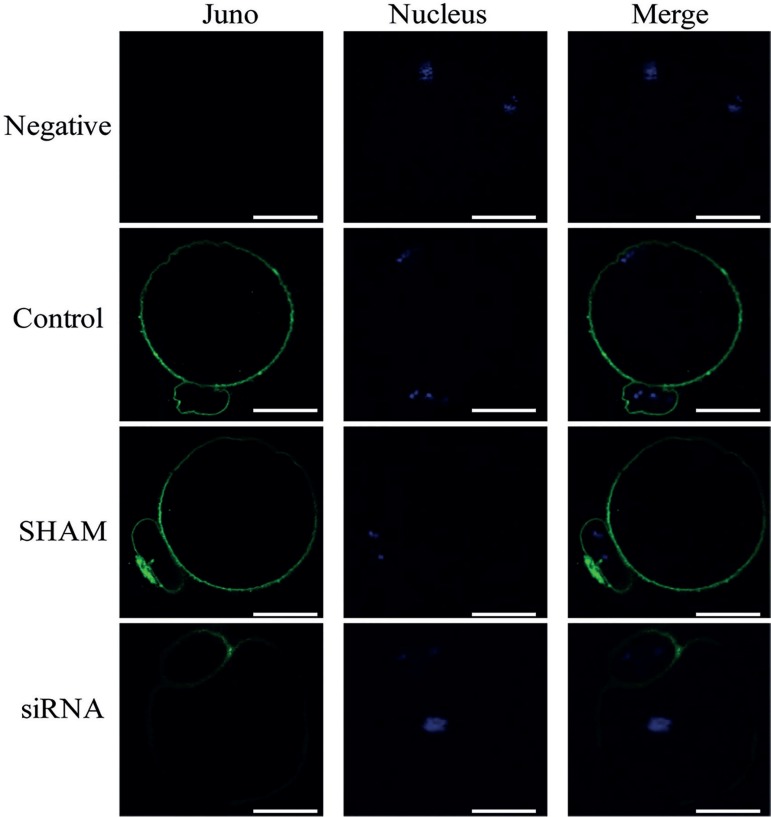
Immunofluorescence staining of MII stage oocyte after siRNA microinjection during GV stage. Juno was stained in green and nucleus was in blue. Scale bar=25 µm

Juno knockdown oocytes were generated by microinjecting 10 nM, 30 nM, or 50 nM siRNA (targeting the Juno mRNA) into the GV stage oocytes. The decrease in Juno protein expression in oolemma was confirmed by immunofluorescence. After siRNA microinjection, weak fluorescence was observed in 59.5% (10 nM siRNA), 64.3% (30 nM siRNA), and 55.0% (50 nM siRNA) oocytes, while 93.5% oocytes displayed strong fluorescence in Sham controls (Table 1). Juno expression was reduced but did not completely disappear in such oocytes (Figure 5).

**Table 1:** Juno fluorescence expression in the cytoplasmic membrane of MII stage oocytes, in which different concentrations of siRNA were microinjected at the GV stage

Concentration of siRNA	Fluorescence pattern		
	Strong (%)	Weak (%)	Total
0 nM (Sham)	44 (93.5)^a^	2 ( 6.5)^a^	46
10 nM	15 (40.5)^b^	22 (59.5)^b^	37
30 nM	15 (35.7)^b^	27 (64.3)^b^	42
50 nM	18 (45.0)^b^	22 (55.0)^b^	40

Values with different superscripts within each column are significantly different (*p*<0.05).

We also analyzed the maturation rate of GV oocytes after siRNA microinjection (Table 2). The 12.0% (10 nM), 10.9% (30 nM) and 12.1% (50 nM) for GV stage; 28.7%, 30.7%, and 32.7% for GVBD-MI stage; and 34.3%, 41.6%, and 37.4% were MII stage, respectively. These values were not significantly different from Sham controls (7.7%, 36.5%, and 44.2% for each stage). Treatment with siRNA at the concentration of 30 nM showed maximum reduction in Juno protein expression and maximum number of MII stage oocytes.

**Table 2:** The rate of maturation at the GV stage in the oocytes microinjected with different concentrations of siRNA

Concentration of siRNA	Oocyte maturation stages				Total
	GV (%)	MI-TI (%)	MII (%)	Flag (%)	
0 nM (Sham)	8 (7.7)	38 (36.5)	46 (44.2)	12 (11.5)	104
10 nM	13 (12.0)	31 (28.7)	37 (34.3)	17 (15.7)	108
30 nM	11 (10.9)	31 (30.7)	42 (41.6)	17 (16.8)	101
50 nM	13 (12.1)	35 (32.7)	40 (37.4)	19 (17.8)	107

Values are not significantly different (*p*<0.05).

## DISCUSSION

Juno is a crucial factor on the oolemma, that recognizes Izumo1 on the sperm surface, to establish oocyte-sperm adhesion (Bianchi *et al*., 2014). In the present study, we demonstrated that Juno is expressed on the oolemma in oocytes at the GV stage, and it continues to be expressed at similar levels during the GVBD-TI and MII stages. Interestingly, we also found Juno expression in the first polar body membrane at the MII stage. Fluorescence showing the expression of Juno was decreased in the oolemma of siRNA injected oocytes, but it was not completely disappeared in knock down oocytes. MII stage-rates of siRNA injected oocytes were not significantly different from sham controls.

In mice, oocytes with a diameter in the range of 15-20 µm can adhere to sperm. However, oocyte-sperm fusion can only occur when the oocyte diameter reaches 20 µm (Zuccotti *et al*., 1994). This suggests that the immature primary oocytes attain the ability to bind to sperm before acquiring the ability to fuse with sperm. Like Juno, CD9 on the oocyte membrane is also an essential factor for oocyte-sperm fusion. In mice, Juno expression begins when the oocyte grows to 13-22 µm in diameter, showing the time when oocytes get the ability to fuse with sperm (Zuccotti *et al*., 1994; Komorowski *et al*., 2006). This points to the possibility that Juno expression either begins at the same time or prior to CD9 expression. Further experiments are required to clarify weather Juno expression occurs even in oogonium. We observed that Juno accumulates in oolemma during the early stage of maturation, which is essential for normal sperm binding.

In this study, we found Juno expression in oocyte oolemma throughout the maturation; however, localization of detailed Juno on membrane was not analyzed. In the oolemma, CD9 is abundantly present in the microvillar rich region, where sperm adhesion and fusion occurs (Zuccotti *et al*., 1994). Since Juno is essential for sperm adhesion, there is a possibility that it also localizes to microvilli. Further, electron microscopic observations are required to determine if Juno accumulates on the microvillar rich region and is involved in microvillus morphogenesis.

Recently, we reported that the images of spindles combined with those of first polar body enable the evaluation and prediction of oocyte and/or embryonic quality (Sugano *et al*., 2016). In this study, Juno expression on the first polar body membrane is consistent with the fact that oocytes in MII stages express Juno protein on their oolemma, as the first polar body is extruded at the end of the TI stage with a little membrane and a little cytoplasm from the sibling oocyte (Fabritius *et al*., 2011). Existence of Juno mRNA on the first polar body has not been determined yet, but the first polar body has a similar mRNA expression as its sibling oocytes in mice and human (Reich *et al.*, 2011; Jiao *et al*., 2014). More recently, the sperm receptor on the oocyte membrane was reduced in aged mice oocytes (Dai *et al*., 2017). Therefore, it is possible that Juno could enable the evaluation and prediction of fertility of the oocyte both on the oolemma and the first polar body.

In conclusion, Juno was expressed in oocytes at the GV stage and it continues to be expressed at similar levels in later stages of oocytes maturation. Thus, Juno accumulation in the oocyte oolemma during maturation would be essential to fertilization, such as membrane recognition of both gametes.
